# Synergetic Antimicrobial Effects of Mixtures of Ethiopian Honeys and Ginger Powder Extracts on Standard and Resistant Clinical Bacteria Isolates

**DOI:** 10.1155/2014/562804

**Published:** 2014-03-17

**Authors:** Yalemwork Ewnetu, Wossenseged Lemma, Nega Birhane

**Affiliations:** ^1^Department of Biotechnology, Natural and Computational Sciences Faculty, University of Gondar, Gondar, Ethiopia; ^2^Department of Parasitology, School of Biomedical and Laboratory Sciences, College of Medicine and Health Sciences, University of Gondar, P.O. Box 196, Gondar, Ethiopia

## Abstract

*Purpose*. To evaluate antimicrobial effects of mixtures of Ethiopian honeys and ginger rhizome powder extracts on *Staphylococcus aureus* (ATCC 25923), *Escherichia coli* (ATCC 25922), *Staphylococcus aureus* (MRSA), *Escherichia coli* (R), and *Klebsiella pneumoniae* (R). *Methods*. Agar diffusion and broth assays were performed to determine susceptibility of these standard and resistant clinical bacteria isolates using honey-ginger powder extract mixtures. *Results*. Honey-ginger powder extract mixtures produced the highest mean inhibition (25.62 mm ± 2.55) compared to the use of honeys (21.63 mm ± 3.30) or ginger extracts (19.23 mm ± 3.42) individually. The ranges of inhibitions produced by honey-ginger extract mixtures on susceptible test organisms (26–30 mm) and resistant strains (range: 19–27 mm) were higher compared to 7–22 mm and 0–14 mm by standard antibiotic discs. Minimum inhibitory concentrations (MIC) of mixture of honeys-ginger extracts were 6.25% (0.625 v/mL) on the susceptible bacteria compared to 75% for resistant clinical isolates. Minimum bactericidal concentration (MBC) of honey-ginger extracts was 12.5% (0.125 g/mL) for all the test organisms. *Conclusion*. The result of this study showed that honey-ginger powder extract mixtures have the potential to serve as cheap source of antibacterial agents especially for the drug resistant bacteria strains.

## 1. Introduction

Plants have been used as a source of therapeutic agents in traditional medicinal system since ancient time due to bioactive compounds they contain [1, 2]. The world health organization (WHO) has described traditional medicine as cheap way to achieve total health care coverage of the world's population and has encouraged the rational use of plant based traditional medicines by member states [3, 4]. In Ethiopia, one of the developing countries, about 80% of the total population relies on traditional remedies as a primary source of health care [3, 5]. Recently, indiscriminate use of antimicrobial drugs to treat the infectious diseases resulted in the development of resistant pathogenic bacteria strains like drug resistant* Escherichia coli*,* Klebsiella pneumonia*, and* Staphylococcus aureus* [6]. Multidrug resistant pathogenic bacteria strains in hospitals and community are the main cause of mortality and morbidity [6–8]. The increasing resistance bacteria against the existing antibiotics resulted in many studies to focus on antimicrobial agents derived from plants [9–12]. Traditional medicine has become a form of complementary medicine and holds a great promise as source of effective therapy for multiple drug resistant strains of bacteria [13, 14].

Ginger has several ethnomedicinal and nutritional values as spice and flavoring agents in Ethiopia and elsewhere [15, 16]. In last few decades, gingeris extensively studied for its medicinal properties by advanced scientific techniques and a variety of bioactive compounds such as tannins, flavonoid, glycosides, essential oils, furostanol, spirostanol, saponins, phytosterols, amides, alkaloids have been isolated from the different parts of the plant which were analyzed pharmacologically [9, 10, 17–19]. The plant was reported for antimicrobial [10, 18–22], nephroprotective [22], anti-inflammatory, and immunomodulatory [23] activities. Traditionally, ginger is reported totreatnausea, vomiting, asthma, cough, palpitation, inflammation, and dyspepsia, loss of appetite, constipation, indigestion, and pain in different parts of the world [24]. Similarly, ginger is used, traditionally, for treating common cold, stomachache, cough, fever, and influenza in Ethiopia [25, 26]. Mixtures of ginger rhizome powder and honeys are also used to treat different types of respiratory and gastrointestinal infections in traditional medicine of Ethiopia. The Ethiopian honeys have also been found effective in producing antibacterial effects on susceptible and resistant strains of bacteria from clinical isolates in Ethiopia [27]. The net effect of herbal drug interactions can be additive, synergetic, or antagonistic [28]. The additive and synergetic effects of phytochemicals in fruit and vegetables are responsible for their potent antioxidant and anticancer activities [29]. The combined antibacterial activity of honey-garlic (*Allium sativum*) or fresh ginger leaves or rhizome extract mixtures was reported superior over the use of these antimicrobial agents individually [12, 30]. Drug interactions of the antimicrobial agents contained in mixtures of Ethiopian honeys and dried ginger rhizome powder extracts were not evaluated. Study on the type of interactions of antimicrobial agents in mixtures of honey and ginger rhizome extract would give an insight about the advantage of using mixtures instead of using ginger or honeys individually. The aim of this study was to evaluate antibacterial effects of mixture of Ethiopian honeys (*Apis mellifera* and stingless bees honeys) and ginger powder ethanol/methanol extracts on standard and resistant clinical isolates of* Staphylococcus aureus*,* Escherichia coli*, and* Klebsiella pneumoniae*.

## 2. Materials and Methods

### 2.1. Study Area and Period

Honeys and ginger rhizomes were purchased in September and October, 2012, G.C from Gondar and Tigray regions in Ethiopia and their antimicrobial effects, including honey-ginger powder extract mixtures, were analyzed in biotechnology laboratory in Gondar University from September 20, 2012, to January 1, 2013.

### 2.2. Chemicals and Reagents

Methanol (B. Number A5791), Ethanol (Alpha chemical B Number M120415, India), Chloroform (BDH chemicals Ltd, Poole, lot Number 27710, England), Acetone, and distilled water were among the different chemicals and solvents used during this study.

### 2.3. Test Organisms

Standard and clinical isolates microorganisms such as* Escherichia coli *(ATCC 25922),* Staphylococcus aureus* (ATCC 25923),* Escherichia coli *(R), Methicillin resistance* Staphylococcus aureus *(MRSA), and* Klebsiella pneumonia *(R) were obtained from Gondar University teaching hospital laboratory.

### 2.4. Preparation of Honey-Ginger Extracts Solutions

Ginger rhizomes were washed with tape and distilled water and sliced into uniform pieces using sterile knife before drying in microoven at 37°C for 24 hours. The dried ginger pieces were crushed using electric grinder to obtain ginger powder. Different ginger extracts were obtained by adding 20 g of ginger powder into 100 mL methanol and ethanol as previous study [31]. Fifty percent ginger solution (50% v/v) was obtained by dissolving 1 mL ginger extract in 1 mL. Ginger powder water extract was considered as negative control as no inhibition zones were found in previous study [31]. The Ethiopian honeys were filtered using sterile gauze to get 100% pure honey. Honey-ginger mixtures will be prepared by mixing 1 mL of the ginger extract and 1 mL honey extract which will be diluted in 2 mL distilled water to obtain honey-ginger extract solution (50% v/v).

### 2.5. Preparation of the Mueller Hinton Agar (MHA)

Mueller Hinton agar (lot Number X4225F, oxoid, England) medium was prepared by dissolving 38 g of Mueller Hinton agar in 1000 mL distilled water and boil until complete dissolutions. The solution was sterilized in an autoclave (121°C, 1 bar) for 15 min. The suspension was poured (20 mL) into sterile petri-dishes in the hood to solidify at room temperature.

### 2.6. Preparation of the Nutrient Broth (7146)

After dissolving 8 g nutrient broth powder in one liter of purified water, the mixture was mixed thoroughly to form a clear medium which would be incubated at 35°C for 18–24 hours after the bacterial specimens were inoculated. Turbidity indicates good growth. Nutrient broth culture medium could live longer under refrigeration.

### 2.7. Preparation of Inoculations and Assays of Antibacterial Activities

The inoculation of the bacteria was done by streaking the surface of the plates with swab in a zigzag manner to spread the bacteria until the entire surface was covered. With a previously sterilized cork borer (4 mm) size, wells of equal distance were bored to drop 100 *μ*L of different ginger extracts and mixtures of honey-ginger powder extracts for agar diffusion assays. Hundred microliters (100 *μ*L) of the honey, ginger powder extract, and honey ginger extract mixtures at 50% (v/v) concentration were inoculated into wells of* Escherichia coli *(ATCC 25922),* Staphylococcus aureus* (ATCC 25923),* Escherichia coli *(R),* Staphylococcus aureus *(MRSA), and* Klebsiella pneumonia *(R). The culture plates were incubated at 37°C for 24 h. Inhibition zones were indicated by clear area around the wells which were measured in millimeters by caliper in order to evaluate the degree of susceptibility of the test organisms.

### 2.8. Preparation of 0.5 McFarland Standards and Standardization of Bacteria Concentration

In this study, 0.5 mL of 0.048 M BaCl_2_ (1.175% W/V BaCl_2_·2H_2_O) was added to 99.5 mL of 0.18 M H_2_SO_4_ (1% V/V) with constant stirring to make 0.5 McFarland Standards. The standard was distributed into a screw capped test tube for color comparison of the test inoculums. Hundred microliter (100 *μ*L) bacteria sample from nutrient broth culture media (lot Himedia laboratory, pvt, ltd., India) was added into 5 mL saline and the concentration was adjusted to 1-2 × 10^8^ colony forming unit per milliliter (Cfu/mL) by comparing with McFarland 0.5 standardized.

### 2.9. Minimum Inhibitory Concentration (MIC) and Minimum Bactericidal Concentration (MBC)

Hundred microliter (100 *μ*L) of bacteria samples from nutrient broth culture medium was added into 5 mL saline and the concentration was adjusted to 1-2 × 10^8^ Cfu/mL by comparing with McFarland 0.5 standard with constant stirring before culturing in new broth medium to determine the lowest concentration of antimicrobial agent capable of preventing growth (Minimum inhibitory concentrations (MIC)). The inoculations of* Escherichia coli *(ATCC 25922),* Staphylococcus aureus* (ATCC 25923),* Escherichia coli *(R),* Staphylococcus aureus *(MRSA), and* Klebsiella pneumonia *(R) were done in different nutrient broth medium containing honey, ginger extract solutions, and mixtures honey-ginger extract solution. The tubes were incubated for 20–24 hours at 37°C to observe turbidity (growth) which indicated the MIC of the honey, ginger extract, and honey-ginger extract mixtures on the test organisms. The minimum bactericidal concentrations (MBCs) were determined by subculturing the contents of nutrient broth used for MIC tests on Mueller Hinton agar media using sterile wire loop and making a strike on the media to see bacteria growth after incubating at 37°C for 24 hours. Absence of growth indicated the minimum bactericidal concentrations (MBCs) of the antimicrobial agents.

### 2.10. Drug Susceptibility

Drug susceptibility of standard* Escherichia coli *(ATCC 25922),* Staphylococcus aureus *(ATCC 25923), and resistant clinical isolates* Escherichia coli *(R),* Staphylococcus aureus *(MRSA), and* Klebsiella pneumonia *(R) cultures were determined using methicillin, amoxicillin, and penicillin discs by disc diffusion method. The result was interpreted as resistant, intermediate, or susceptible by comparing the results with what has already been reported by Clinical and Laboratory Standards Institute (CLSI) [32].

### 2.11. Statistical Analysis

The antibacterial effects (inhibitions) of honey, ginger extracts, and honey-ginger extracts mixtures were compared using descriptive statistics. All statistical analysis has been performed by using statistical package of social science (SPSS) version 20. Comparisons of honeys, ginger extracts, and honey-ginger extracts mixtures for their overall mean inhibitions were analyzed using one-way analysis of variance (ANOVA). *P* values less than 0.05 were considered as significantly different. Further Tukey's Honestly Significant Difference (HSD) test or post hoc test was performed to see the effect of the antimicrobial agents on the individual bacteria strain.

## 3. Results

**Figure 1:**
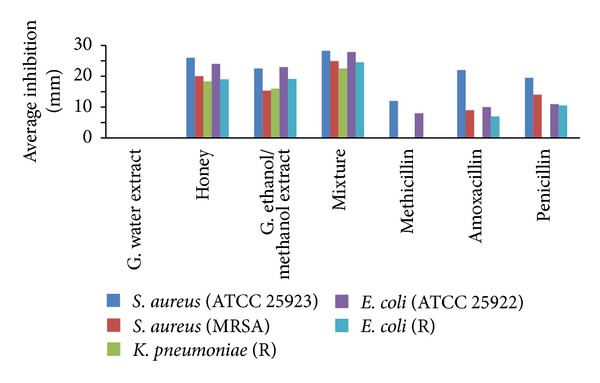
Average inhibition in mm for ginger powder water extracts, honeys, ginger powder ethanol/methanol extracts, mixture of honeys-ginger extracts, and standard antibiotic discs (Methicillin, Amoxicillin, and Penicillin).

The overall comparison of the antimicrobial agents has shown that honey-ginger extract mixtures produced the highest mean inhibition (25.62 mm ± 2.55) for the total test organisms compared to the use of honeys (21.63 mm ± 3.30) or ginger extracts (19.23 mm ± 3.42) individually. The least mean inhibition result was obtained from the Methicillin antibiotic discs (4 mm ± 5.26) (Table 1). Mean inhibition of the honey-ginger extract mixture on susceptible bacteria isolates (27.74 mm ± 1.56) was higher compared to its effect on resistant isolates (23.97 mm ± 1.72). Similarly, the average inhibition of the honeys, ginger extracts, and honey-ginger extract mixtures was higher on susceptible bacteria strains compared to resistant clinical isolates (Figure 1). Ginger water extract did not produce any inhibition indicating the bioactive agents in the ginger powder are not water soluble. The overall mean inhibition of honeys of stingless bees and* Apis mellifera* was the same as statistical analysis using ANOVA and Tukey's Honestly Significant Difference (HSD) test (multiple comparison) which was greater than 0.05 (*P* > 0.05). Similarly, ginger extracts using methanol and ethanol solvents showed no differences for their inhibitions on the test bacteria strains. But statistically significant difference was observed when overall mean inhibitions of honeys, ginger extracts, and honey-ginger extract mixtures were compared (*P* = 0.00) (Table 1).

The highest inhibition (30 mm) was produced by honey-ginger extract mixtures on the susceptible bacteria strains. The range of inhibitions produced by honey-ginger extract mixtures on the susceptible bacteria isolates (26–30 mm) and resistant clinical isolates (19–27 mm) was also greater than 7–22 mm on susceptible or 0–14 mm on resistant isolates produced by the antibiotic discs (Methicillin, Amoxicillin, and Penicillin) (Table 1). When mean inhibition results on susceptible and resistant bacteria isolates were compared using ANOVA, there were statistically significant differences (*P* = 0.000) for honey, ginger extract, and honey-ginger extract mixture (Table 1).

Further Tukey's Honestly Significant Difference (HSD) test or post hoc test, however, showed absence of significant difference between mean inhibitions of susceptible on* S. aureus *(ATCC 25923) and* E. coli* (ATCC 25922) when honey (*P* = 0.964), ginger extract (*P* = 0.964), and honey-ginger mixture (*P* = 0.973) were used (Table 2). Similarly, no differences in mean inhibitions were found when the results between the resistant* S. aureus *(MRSA) and* E. coli* (R) were compared for honey-ginger mixture alone (*P* = 0.940).

The minimum bactericidal concentration (MBC) for honeys, ginger extracts, and honey-ginger extract mixtures was 12.5% (0.125 g/mL). The bactericidal effect of this concentration was 100% for all test organisms. The minimum inhibitory concentrations (MIC) of honeys, ginger extracts, and honey-ginger extract mixtures were shown in Table 3. Honey-ginger extract mixtures have 6.25% (0.0625 g/mL) MIC values for all susceptible strains compared to 75% for resistant clinical isolates (Table 3).

## 4. Discussion

The ginger powder water extract (negative control) did not show bacterial growth inhibition on the test organisms (Figure 1) as it has already been reported [31]. But higher inhibition results were found for ginger ethanol or methanol extracts (19.23 mm ± 3.42) on the test organisms. The highest inhibition (24 mm) produced by this ginger powder extract on* S. aureus* (ATCC 25923) (Table 1) was less than 30 mm for* S. aureus *using fresh ginger rhizome ethanol extract in similar study [11]. This difference could be explained by the loss of water soluble antioxidant volatile oils from the ginger powder up on dehydration [22, 33]. Despite the loss of some antibacterial agents by evaporation during making ginger powder [33], antibacterial agents extracted by the organic solvents were enough to produce inhibition on both susceptible* S. aureus *ATCC 25923 and* E. coli* ATCC 25922 (24 mm) that was greater than inhibitions produced by the three positive control antibiotic discs (range: 7–22 mm) (Table 1). According to Clinical and Laboratory Standards Institute (CLSI) [32] standardization of antibiotic discs, inhibition is considered susceptible if it is ≥18 mm for Amoxicillin (25 *μ*g), ≥14 mm for Methicillin (5 *μ*g), and ≥29 mm for Penicillin (10 *μ*g). The results obtained from amoxicillin discs showed* S. aureus *(ATCC 25923) to be susceptible as already reported by standardization manual [32]. Very low inhibition results from standard antibiotic discs identified the resistant clinical isolates. The mean (23.97 mm ± 1.72) and range (19–27 mm) of inhibitions produced by honey-ginger extract mixtures on resistant clinical isolates were greater than mean (6.38 mm ± 5.24) and range (0–14 mm) of antibiotic discs (Methicillin, Amoxicillin, and Penicillin). The inhibitions of ginger powder ethanol/methanol extracts were enhanced (25.62 mm ± 2.55) by mixing with honeys due to their synergistic antibacterial effects of honey-ginger extract mixtures as already reported [10]. The ginger powder ethanol/methanol extracts were positive for known antimicrobial agents such as saponin, alkaloids, phlobatannin, flavonoids, and cardiac glycosides [10]. The antimicrobial effects of different honeys might be related to Phytochemicals such as Phenolic acids (benzoic and cinnamic acids) and flavonoids (flavanones, flavanols) which were reported for significant contribution of the antioxidant capacity of honey that varies greatly depending on the floral sources [34]. The presence of propolis makes stingless honeybees honey slightly different in antimicrobial effect from* Apis mellifera* honey [35]. Propolis (resinous protective barrier) contains flavonoids, aromatic acids, esters, aldehydes, ketones, fatty acids, terpenes, steroids, amino acids, polysaccharides, hydrocarbons, alcohols, hydroxybenzene, and several other compounds in trace amounts [36]. When* Apis mellifera* honey (eucalyptus) and stingless honeybees honey were compared, the former had higher phenolic and flavonoid contents than the stingless bee honey which in turn had the higher Antioxidant activity [37]. The antimicrobial effects of different Ethiopian honeys were already evaluated with stingless bees honey producing slightly greater mean inhibition compared to* Apis mellifera* honey [27]. But Tukey's Honestly Significant Difference (HSD) test (post hoc test) showed absence of significant difference between mean inhibitions of stingless honeybees honey and* Apis mellifera* honey (*P* = 0.964) on susceptible on* S. aureus *(ATCC 25923) and* E. coli* (ATCC 25922). Many, but not all, of the bacterial strains commonly encountered by humans are killed by flavonoids, even though the mechanism is not yet known [38]. The synergistic antimicrobial effects of honey-ginger extract mixtures might be related chiefly to the increase in volume of these flavonoides in the mixture since both ingredients contained these antimicrobial agents.

Antimicrobial activities (antibacterial, antiviral, antifungal, and antiparasitic) of honeys were reported due to high osmolarity, acidity, hydrogen peroxide, and phytochemicals [39–45].* In vivo* use of honey for human as therapeutic agent depends on the evaluation of the nonperoxide phytochemical components of honey as hydrogen peroxide can be destroyed by catalase in the body tissues and serum [46]. Similarly, the high osmolarity and acidity of honeys are destroyed in the digestion system or blood circulation of human. The nonperoxide phytochemical components of Manuka Apinae honey (after removing hydrogen per oxide by treating with enzyme catalase) from New Zealand have been found to have substantial levels of antibacterial activity [47]. Such manuka honey was tested against seven species of bacteria and was found to have MIC (minimum inhibitory concentration) that range from 1.8% to 10.8% (v/v) [48]. Probably, oral administration of honey-ginger extract mixture, after clinical evaluation and pharmacological standardization, might be therapeutic for some drug resistant disease causing bacteria strains. The minimum bactericidal concentration (MBC) for this study was 12.5% (0.125 g/mL) for both susceptible and resistant bacteria strains. The minimum inhibitory concentrations (MIC) of honey-ginger extract mixture were 6.25% (0.0625 g/mL) for all (100%) susceptible and 75% resistant bacteria strains (Table 3). The fact that both honey and ginger are used in human nutrition and the effectiveness of their mixture as antimicrobial agent at very low concentration make honey-ginger mixture a novel source of effective drug for resistant bacteria strains. Further clinical evaluation and pharmacological standardization of honey-ginger extract mixtures are recommended before using the mixtures against drug resistant bacteria strains for therapeutic purposes.

## 5. Conclusion

In conclusion, honeys-ginger powder extract mixtures were found to have more antimicrobial effect than the use of honeys or ginger extracts solutions individually. The use of honeys and ginger extracts mixtures for drug resistant bacteria such as* staphylococcus aureus *(MRSA),* Escherichia coli *(R), and* Klebsiella pneumonia *(R) is recommended.

## Figures and Tables

**Table 1:** Results of one-way ANOVA and descriptive statistics of the inhibitions of honeys, ginger extracts, and honey-ginger extracts mixtures on the test bacteria isolates.

Agents	Test organisms	*N*	Mean	Standard deviation	Minimum	Maximum
Honey*	*Staphylococcus aureus* (ATCC 25923)	6	22.50	1.64	21.00	24.00
	*Staphylococcus aureus* (MRSA)	6	15.50	1.517	13.00	17.00
	*Klebsiella pneumoniae*(R)	6	16.00	0.63	15.00	17.00
	*Escherichia coli* (ATCC 25922)	6	23.00	1.27	21.00	24.00
	*Escherichia coli* (R)	6	19.17	1.33	18.00	21.00
	Total	**30**	**19.23**	**3.42**	**13.00**	**24.00**

Extract*	*Staphylococcus aureus* (ATCC 25923)	6	22.50	1.64	21.00	24.00
	*Staphylococcus aureus* (MRSA)	6	15.50	1.52	13.00	17.00
	*Klebsiella pneumoniae* (R)	6	16.00	0.63	15.00	17.00
	*Escherichia coli *(ATCC 25922)	6	23.00	1.26	21.00	24.00
	*Escherichia coli* (R)	6	19.17	1.33	18.00	21.00
	Total	**30**	**19.23 **	**3.42 **	**13.00**	**24.00**

Mixture*	*Staphylococcus aureus* (ATCC 25923)	12	28.25	1.14	27.00	30.00
	*Staphylococcus aureus* (MRSA)	12	24.92	1.56	22.00	27.00
	*Klebsiella pneumoniae* (R)	12	22.50	1.57	19.00	25.00
	*Escherichia coli *(ATCC 25922)	12	27.92	1.38	26.00	30.00
	*Escherichia coli* (R)	12	24.50	0.90	23.00	26.00
	Total	** 60**	**25.62**	**2.55**	**19.00**	**30.00**

Methicillin*	*Staphylococcus aureus* (ATCC 25923)	3	12.00	2.55	11.00	13.00
	*Staphylococcus aureus* (MRSA)	3	0.00	1.00	0.00	0.00
	*Klebsiella pneumoniae* (R)	3	0.00	0.00	0.00	0.00
	*Escherichia coli *(ATCC 25922)	3	8.00	0.00	7.00	9.00
	*Escherichia coli* (R)	3	0.00	1.00	0.00	0.00
	Total	**12**	**4.00 **	**0.00 **	**0.00**	**13.00**

Amoxicillin*	*Staphylococcus aureus* (ATCC 25923)	3	21.00	5.26	20.00	22.00
	*Staphylococcus aureus* (MRSA)	3	10.00	1.00	9.00	11.00
	*Klebsiella pneumoniae* (R)	3	—	1.00	—	—
	*Escherichia coli *(ATCC 25922)	3	10.00	—	10.00	10.00
	*Escherichia coli* (R)	3	8.00	0.00	7.00	9.00
	Total	**12**	**12.25**	**1.00**	**7.00**	**22.00**

Penicillin*	*Staphylococcus aureus* (ATCC 25923)	3	20.00	5.40	19.50	20.50
	*Staphylococcus aureus* (MRSA)	3	13.00	0.50	12.00	14.00
	*Klebsiella pneumoniae* (R)	3	—	1.00	—	—
	*Escherichia coli *(ATCC 25922)	3	10.00	—	9.00	11.00
	*Escherichia coli* (R)	3	10.00	1.00	9.50	10.50
	Total	**12**	**13.25**	**0.50**	**9.00**	**20.50**
				4.32		

*The mean difference is significant at the 0.05 level.

**Table 2:** Results of Tukey's Honestly Significant Difference (HSD) test of honey-ginger extract mixtures on the test bacteria isolates.

Post hoc test	(*I*) spp.	(*J*) spp.	Mean difference (*I* − *J*)	Std error	Sig
Mixture	*Staphylococcus aureus* (ATCC 25923)	*Staphylococcus aureus* (MRSA)	3.33333*	0.54518	0.000
		*Klebsiella pneumoniae *(R)	5.75000*	0.54518	0.000
		*Escherichia coli * (ATCC 25922)	0.33333	0.54518	0.973
		*Escherichia coli *(R)	3.75000*	0.54518	0.000
	*Staphylococcus aureus* (MRSA)	*Staphylococcus aureus* (ATCC 25923)	−3.33333*	0.54518	0.000
		*Klebsiella pneumoniae *(R)	2.41667*	0.54518	0.000
		*Escherichia coli* (ATCC 25922)	−3.00000*	0.54518	0.000
		*Escherichia coli* (R)	0.41667	0.54518	0.940
	*Klebsiella pneumoniae *(R)	*Staphylococcus aureus* (ATCC 25923)	−5.75000*	0.54518	0.000
		*Staphylococcus aureus* (MRSA)	−2.41667*	0.54518	0.000
		*Escherichia coli* (ATCC 25922)	−5.41667*	0.54518	0.000
		*Escherichia coli* (R)	−2.00000*	0.54518	0.005
	*Escherichia coli* (ATCC 25922)	*Staphylococcus aureus* (ATCC 25923)	−0.33333	0.54518	0.973
		*Staphylococcus aureus* (MRSA)	3.00000*	0.54518	0.000
		*Klebsiella pneumoniae *(R)	5.41667*	0.54518	0.000
		*Escherichia coli* (R)	3.41667*	0.54518	0.000
	*Escherichia coli* (R)	*Staphylococcus aureus* (ATCC 25923)	−3.75000*	0.54518	0.000
		*Staphylococcus aureus* (MRSA)	−0.41667	0.54518	0.940
		*Klebsiella pneumoniae *(R)	2.00000*	0.54518	0.005
		*Escherichia coli* (ATCC 25922)	−3.41667*	0.54518	0.000

*The mean difference is significant at the 0.05 level.

**Table 3:** Minimum inhibitory concentration (MIC) of honey, ginger extracts, and honey-ginger extract mixtures on susceptible and resistant isolates of the test bacteria species.

Susceptible bacteria isolate	Honey	G. extract	Mixture	Resistant clinical isolates	Honey	G. extract	Mixture
*S. aureus * (ATCC 25923)	6.25%	6.25%	6.25%	*S. aureus *(MRSA)	12.5%	12.5%	6.25% & 12.5%
*E. coli* (ATCC 25922)	6.25%	6.25% & 12.5%	6.25%	*E. coli* (R)	6.25% & 12.5%	12.5%	6.25% & 12.5%
				*K. pneumoniae *(R)	6.25% & 12.5%	12.5%	6.25% & 12.5%

at 6.25% (0.0625 gm/mL)	100%	75%	100%		60%	0%	75%
% at 12.50% (0.125 gm/mL)	0 %	25%	0 %		40%	100%	25%
